# Cannabinoids and Terpenes: How Production of Photo-Protectants Can Be Manipulated to Enhance *Cannabis sativa* L. Phytochemistry

**DOI:** 10.3389/fpls.2021.620021

**Published:** 2021-05-31

**Authors:** Vincent Desaulniers Brousseau, Bo-Sen Wu, Sarah MacPherson, Victorio Morello, Mark Lefsrud

**Affiliations:** Department of Bioresource Engineering, McGill University, Sainte-Anne-de-Bellevue, QC, Canada

**Keywords:** light emitting diode, light spectrum, light wavelength, photobiology, secondary metabolites, tetrahydrocannabinol, ultraviolet

## Abstract

*Cannabis sativa* L. is cultivated for its secondary metabolites, of which the cannabinoids have documented health benefits and growing pharmaceutical potential. Recent legal cannabis production in North America and Europe has been accompanied by an increase in reported findings for optimization of naturally occurring and synthetic cannabinoid production. Of the many environmental cues that can be manipulated during plant growth in controlled environments, cannabis cultivation with different lighting spectra indicates differential production and accumulation of medically important cannabinoids, including Δ^9^-tetrahydrocannabinol (Δ^9^-THC), cannabidiol (CBD), and cannabigerol (CBG), as well as terpenes and flavonoids. Ultraviolet (UV) radiation shows potential in stimulating cannabinoid biosynthesis in cannabis trichomes and pre-harvest or post-harvest UV treatment merits further exploration to determine if plant secondary metabolite accumulation could be enhanced in this manner. Visible LED light can augment THC and terpene accumulation, but not CBD. Well-designed experiments with light wavelengths other than blue and red light will provide more insight into light-dependent regulatory and molecular pathways in cannabis. Lighting strategies such as subcanopy lighting and varied light spectra at different developmental stages can lower energy consumption and optimize cannabis PSM production. Although evidence demonstrates that secondary metabolites in cannabis may be modulated by the light spectrum like other plant species, several questions remain for cannabinoid production pathways in this fast-paced and growing industry. In summarizing recent research progress on light spectra and secondary metabolites in cannabis, along with pertinent light responses in model plant species, future research directions are presented.

## Introduction

Secondary metabolites from plants, animals, and microorganisms drive many medical and pharmacological applications, building on thousands of years of traditional medicine (Stojanoski, 1999). In depth characterization of isolated plant secondary metabolites (PSM) for medical treatment started at least 200 years ago, and it has progressed exponentially during the last 30 to 40 years (Okada et al., 2010). One notable and historical medical application is the isolation of morphine from poppy (*Papaver somniferum*) seed oil in the early 1800s (Krishnamurti and Rao, 2016). This alkaloid and its derivatives, opiates, are used for managing pain, yet they have contributed to a deadly and costly opioid crisis because of their addictive nature (Dasgupta et al., 2018).

The cannabis plant (*Cannabis sativa* L.) possesses more than 500 known PSM, including cannabinoids, terpenes, and flavonoids (Elsohly et al., 2017; Solymosi and Köfalvi, 2017; Gonçalves et al., 2019). Research on cannabis PSM has grown rapidly because of therapeutic potential. The cannabinoid Δ^9^-tetrahydrocannabinol (Δ^9^-THC), a hallmark of medical cannabis, reportedly exerts anticancer (White et al., 1976), antibacterial (Van Klingeren and Ten Ham, 1976), antiemetic (Garb, 1981), and analgesic action *via* modulation of the endocannabinoid system (Mao et al., 2000), and it remains a possible alternative to opiates for managing neuropathies and treatment-resistant spasticity (Abrams, 2019). Specific cannabinoid-terpenoid ratios from herbal extracts have shown further promise (Gonçalves et al., 2019), and provide support for the “entourage effect,” the postulated synergistic action of cannabinoids and terpenes with notable examples in pain management (Johnson et al., 2010), analgesia (Gallily et al., 2015), cancer (Blasco-Benito et al., 2018), and severe epilepsy (Goldstein, 2016).

Prior to cannabis legalization, our knowledge of cannabis PSM production primarily stemmed from illegal production operations (Vanhove et al., 2011). Over the last few years, enormous progress has been made toward advancing cannabis-related medicine (Hutchison et al., 2019) and cannabis biotechnology (i.e., productivity and molecular biology) (Hesami et al., 2020). Phytochemical characterization of a given cultivar (or the newly coined term “chemovar”), including biochemical and pharmacological properties, could drive this next era of medicine forward (Russo, 2019), but thorough understanding of cannabis PSM production and accumulation mechanisms are required. Contemporary medicine highlights cannabinoids, terpenes, and flavonoids as promising PSMs for treating multiple ailments (Aliferis and Bernard-Perron, 2020).

Evidence suggests that growing conditions (i.e., light, nutrients, temperature, and microbiome) can be manipulated to improve and optimize production of specific compounds. Light triggers plant secondary metabolism and PSM accumulation, although how optical and spectral properties (i.e., wavelength, bandwidth, and intensity) impact cannabis PSM production remains unclear (Hawley, 2018; Magagnini et al., 2018; Namdar et al., 2019). This review aims to bridge the gap between light properties and cannabis PSM production, by recalling PSM origin and function in plants. An overview of the cannabis PSM biosynthesis, including cannabinoid, terpene, and flavonoid, is provided in the support of the “entourage effect” (Baron, 2018; Tomko et al., 2020). Available light study findings on cannabis PSM production in response to different light treatments are summarized, with an emphasis on ultraviolet (UV) radiation during plant growth.

## Evolutive Perspective of Plant Secondary Metabolites

PSM are assembled from primary metabolite precursors (Seca and Pinto, 2019). These PSM are not essential to plants' survival; rather, they allow plants to withstand abiotic and biotic stress (drought or water stress, light or predatory stress) (Bourgaud et al., 2001). PSM molecular pathways are conserved between plant families through gene clusters. Genome sequencing has shown that these gene clusters are highly conserved between plants of different families because of their shared evolutive origin (Nützmann et al., 2016). In *C. sativa* L., cannabinoid and terpene biosynthesis reportedly contributes to protection against UV radiation and chemical stressors created to combat insects (Pate, 1994; Benelli et al., 2018).

PSM likely evolved in an environment where biotic stressors played a lesser role in driving evolutive adaptation (Tossi et al., 2019). By looking at other PSM functions and their role in the plant's response to abiotic stress, one theory states that to survive in shallow water, ancestral algae evolved mechanisms to survive in an environment with elevated UV radiation (<380 nm), a primordial abiotic stressor (Akula and Ravishankar, 2011; Jenkins, 2017). UV radiation leads to damaged DNA and photosystems, resulting in reduced production (Teramura, 1983). Plants evolved mechanisms to protect against this radiation stress by accumulating phenolic and terpenoid compounds that absorb UV radiation and acted as sunscreen in leaves (Rozema et al., 2002). This allowed photosynthetic organisms to grow in new ecological niches, while exposing themselves to increasing UV radiation (Tossi et al., 2019). This theory is supported by the apparition of a highly conserved receptor, UV-B Resistance 8 (UVR8) in terrestrial plants that mediates plant photomorphogenesis in response to UV radiation (Jenkins, 2017; Tossi et al., 2019). Parallel to the abiotic stress response, the large diversity of PSM can also be explained by exposure to biotic stress and the co-evolution of insects and plants during terrestrialization in the Neoproterozoic era (1,000 to 541 million years ago) (Theis and Lerdau, 2003; Labandeira, 2005). Plants evolved attractant and deterrent cues through their PSM to favor pollination and decrease predation (Kessler and Halitschke, 2007). Studies report that cannabis PSM extracts, specifically hemp extracts, effectively repel insects (Mcpartland, 1997; Benelli et al., 2018). The cannabis microbiome also influences plant metabolism. A recent review highlights promising avenues of PSM modulation in cannabis through endophytes (Taghinasab and Jabaji, 2020).

## Trichomes And Cannabis Profiling

### Trichomes

Trichomes form a large group of plant structures that are uni- or multicellular epidermal appendages, classified by their origin, form, function, and secretion (Werker, 2000). These structures are responsible for synthesis and storage of cannabinoids and terpenes in *C. sativa* L., accumulating in resin heads (Hudson, 1963). They protect plants from light stress (Lydon et al., 1987), high heat (Levin, 1973; Lapinjoki et al., 1991), and herbivore pressure (Pillemer and Tingey, 1976; Alahakoon et al., 2016). Other mechanisms, including water absorption through dew collection, salt secretion, and alluring function, are reported (Werker, 2000).

All aerial parts of the cannabis plant are covered with trichomes, and can be classified as either “glandular” or “non-glandular” (Dayanandan and Kaufman, 1976). Glandular trichomes contain more bioactive/psychoactive compounds than non-glandular trichomes (Raman et al., 2017; Livingston et al., 2020). Glandular trichomes are found on all anatomical plant parts except the hypocotyl and cotyledon, and non-glandular trichomes are found on stems, leaves, petioles, stipules, bract, and tepals (Raman et al., 2017).

Glandular trichome classification relates to morphological traits and composition of the chemical substance secreted (Werker, 2000). Three types of glandular trichomes in the cannabis plant are described and size-differentiated: capitate-stalked, capitate-sessile, and bulbous trichomes (Dayanandan and Kaufman, 1976; Hammond and Mahlberg, 1977). Capitate-stalked trichomes are found exclusively on flowering regions, whereas capitate-sessile and bulbous trichomes are found everywhere except the hypocotyl and cotyledon (Raman et al., 2017). In *C. sativa* L., high THC-containing strains had a bigger resin head on their glandular trichomes than in low-THC industrial hemp (Small and Naraine, 2016). Capitate-stalked glandular trichomes have more secretory disc cells than other plants and secrete specialized metabolites in the subcuticular oil storage cavity, instead of through pores formed in the cuticle (Tissier, 2012; Huchelmann et al., 2017). Excretory cells secrete a resin in a subcuticular cavity (Small and Naraine, 2016). This resin contains high concentrations of the economically important cannabinoids, with psychoactive and medicinal properties (Dayanandan and Kaufman, 1976; Small and Naraine, 2016). Optimal cannabinoid and terpene biosynthesis in glandular trichomes is of paramount importance to bud quality (El-Alfy et al., 2010; Friedman and Devinsky, 2015).

### Cannabinoids and Cannabis Profiling

Cannabinoids, also called meroterpenes or terpenophenols, are PSM synthesized by members of the *Cannabaceae* family, and several other plant species, including *Echinacea purpurea, Echinacea angustifolia, Acmella oleracea, Helichrysum umbraculigerum*, and *Radula marginata* (Bauer et al., 2008). More than 20% of isolated cannabis PSMs are cannabinoids (Chandra et al., 2017). The two major cannabinoids, Δ^9^-THC and cannabidiol (CBD), are used to classify cannabis (Bruci et al., 2012; Piluzza et al., 2013; Hilderbrand, 2018), and differentiation between marijuana and hemp is often based on Δ^9^-THC content from cannabis biomass. Cannabis extract with a Δ^9^-THC percentage greater than 0.3% is classified as a medical marijuana product, whereas *C. sativa* L. with a Δ^9^-THC content of less than 0.3% is cultivated as hemp (Hilderbrand, 2018). Three *C. sativa* L. chemotypes have further been distinguished and classified, determined by the relative proportions of Δ^9^-THC and CBD: drug-type (Δ^9^-THC is the predominant cannabinoid, known as marijuana), intermediate-type (both Δ^9^-THC and CBD are predominant), and fiber-type (CBD is the predominant cannabinoid, known as hemp) (Bruci et al., 2012; Piluzza et al., 2013). This differentiation based on cannabinoid content or cannabis cultivars is inadequate, particularly for the medical industry, since it does not reflect or match the therapeutic and medical properties (Russo, 2019). The term “chemovar,” which considers the specific ratios of cannabinoids, flavonoids, and terpenes, will likely be a better tool in the development of cannabis-assisted medicine (Baron, 2018).

## Cannabis PSMs and Biosynthesis

Changes in PSM biosynthesis during ontological development of cannabis are well-studied, starting with cannabinoid and monoterpene concentrations in flowers in the first weeks of the flowering phase, and ending with almost four times the quantity in a matter of 7 weeks (Aizpurua-Olaizola et al., 2016). At least 113 cannabinoids and 120 terpenes have been identified (Elsohly and Slade, 2005; Elsohly and Gul, 2014; Ahmed et al., 2015), and they are heavily concentrated in virgin female inflorescence (Turner et al., 1980). PSMs are usually extracted from this, as maximal PSM accumulation is often found in glandular trichomes. Other studies have concentrated on determining the role that flavonoids play in cannabis physiology, and how cannabis-specific flavonoids may be exploited (Barrett et al., 1985; Pollastro et al., 2018).

### Cannabinoids

**Figure 1:**
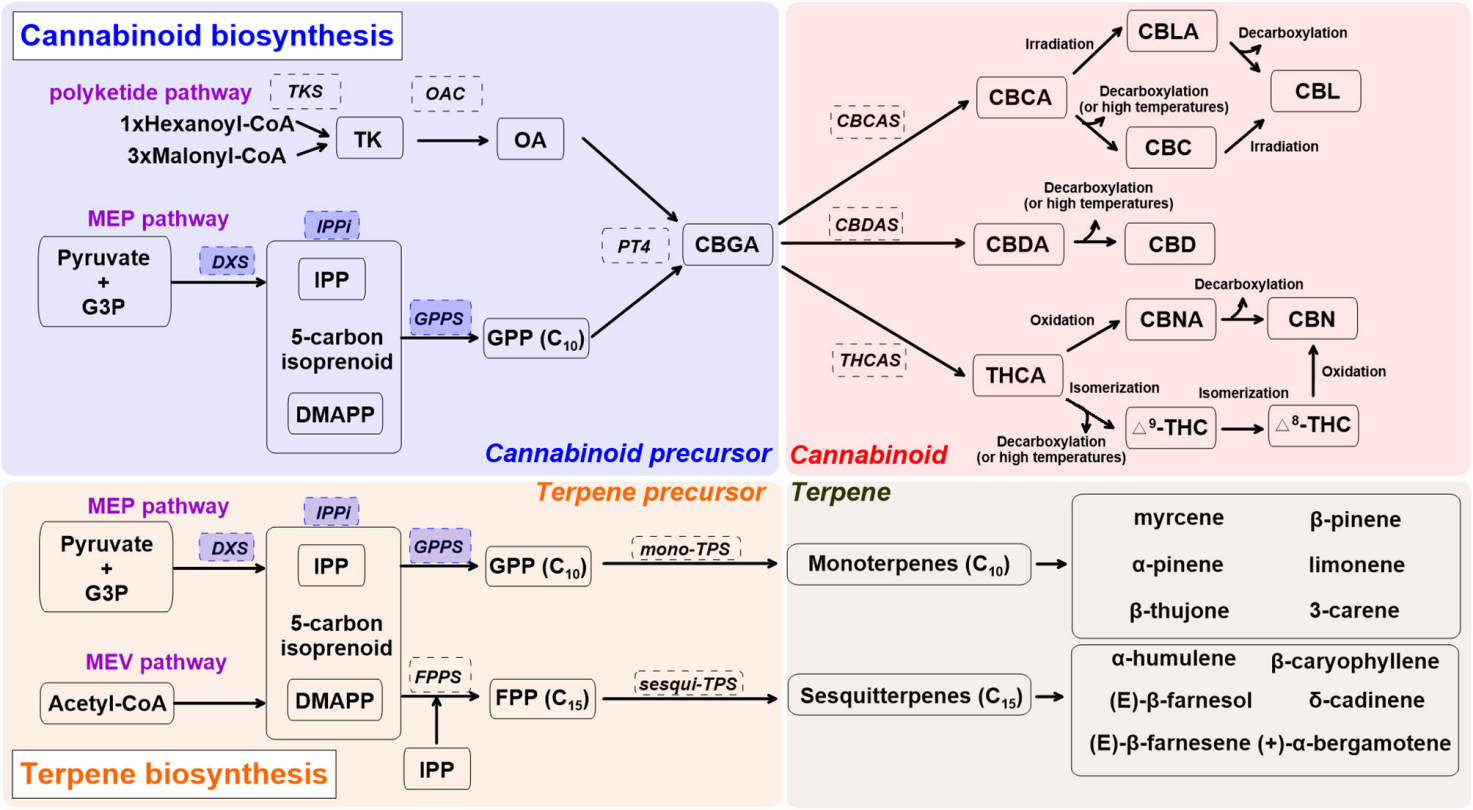
A simplified overview of cannabinoid and terpene biosynthesis pathways in cannabis (*Cannabis sativa* L.), derived from recent reviews (Hazekamp, 2007; Degenhardt et al., 2017; Sirikantaramas and Taura, 2017; Jin et al., 2019). Enzymes are in dashed line box. Enzymes in shaded blue boxes are upregulated by UV radiation and blue light in Lamiaceae. Cannabis precursor (shade blue): CBDA, cannabidiolic acid; DMAPP, dimethylallyl pyrophosphate; G3P, glyceraldehyde 3-phosphate; GPP, geranyl pyrophosphate; GPPS, geranyl pyrophosphate synthase; MEP, methylerythritol phosphate; PT4, geranylpyrophosphate: olivetolate geranyltransferase 4; IPP, isopentenyl diphosphate; IPPi, isopentenyl-diphosphate delta-isomerase; OA, olivetolic acid; OAC, olivetolic acid cyclase; TK, tetraketide; TKS, tetraketide synthase. Cannabinoid (shade red): CBC, cannabichromene; CBCA, cannabichromentic acid; CBCAS, cannabichromentic acid synthase; CBDAS, cannabidiolic acid synthase; CBD, cannabidiol; CBG, cannabigerol; CBGA, cannabigerolic acid; CBL, cannabicyclol; CBLA, cannabicyclolic acid; CBN, cannabinol; CBNA: cannabinolic acid; Δ^8^-THC, Δ^8^-tetrahydrocannabinol; Δ^9^-THC (or THC), Δ^9^-tetrahydrocannabinol; THCA, tetrahydrocannabinolic acid. Terpene precursor (shade orange): FPP, farnesyl diphosphate; FPPS, farnesyl diphosphate synthase; MEV, mevalonate; TPS, terpene synthase.

Figure 1 shows the cannabinoid biosynthesis pathway and precursor formation. Primary biosynthesis steps are impacted by UV radiation and blue light (Dolzhenko et al., 2010; Booth et al., 2017; Jin et al., 2019; Nazari and Zarinkamar, 2020). Cannabinoid biosynthesis starts as isopentenyl diphosphate (IPP), formed from glyceraldehyde 3-phosphate (G3P), and pyruvate in plastids (Mcgarvey and Croteau, 1995). Formation of IPP in plastids is ensured by 1-deoxy d-xylulose-5-phosphate synthase (DXS), part of the methylerythritol phosphate (MEP) pathway (Lichtenthaler, 1999). The 5-carbon isoprenoid then is linked with isopentenyl diphosphate (IPP) and dimethylallyl pyrophosphate (DMAPP) through isopentenyl-diphosphate delta-isomerase (IPPi). These are condensed into geranyl diphosphate (GPP, C_10_) *via* GPP synthase (GPPS) (Ruzicka, 1953; Hunter, 2007). GPP also acts as a precursor for monoterpene biosynthesis. The enzymes DXS, IPPi, and GPPS are upregulated by UV radiation and blue light in peppermint (*Mentha x piperita*) and water mint (*Mentha aquatica*) (Dolzhenko et al., 2010; Nazari and Zarinkamar, 2020).

Olivetolic acid (OA) sets cannabinoid and monoterpene biosynthesis apart. It is produced through a type III polyketide synthase, leading to formation of the cannabinoid precursor, cannabigerolic acid (CBGA) (Gagne et al., 2012). The first step of OA formation is ensured by a unique tetraketide synthase (TKS) and olivetolic acid cyclase (OAC) (Luo et al., 2019). This step uses 1 hexanoyl-CoA and 3 malonyl-CoA to form OA *via* a tetraketide (TK) intermediate. OA is then prenylated by geranylpyrophosphate: olivetolate geranyltransferase 4 (PT4) to form the central precursor molecule CBGA, which can then be further modified into constituents such as Δ^9^-THC, CBD, and cannabichromene (CBC) (Flores-Sanchez and Verpoorte, 2008b; Luo et al., 2019). CBGA is converted to cannabidiolic acid (CBDA), tetrahydrocannabinolic acid (THCA), and cannabichromentic acid (CBCA) by cannabidiolic acid synthase (CBDAS), tetrahydrocannabinolic acid synthase (THCAS), and cannabichromentic acid synthase (CBCAS), respectively. During these steps, cannabinoids are naturally converted from their acid forms during storage or heating (decarboxylation) as non-enzymatic catalyzed reactions (Veress et al., 1990). THCA and CBDA reactions are oxygen-dependent and produce hydrogen peroxide, as opposed to the CBCA reaction, which is oxygen-independent and can be inhibited by hydrogen peroxide (Sirikantaramas et al., 2004; Taura et al., 2007; Degenhardt et al., 2017).

CBCA is most actively synthesized in young cannabis seedlings and can be found in both drug-type and fiber-type cannabis plants, yet its concentration is relatively low compared to other cannabinoids (Kushima et al., 1980; Chandra et al., 2017). CBCA is converted to CBC (Gaoni and Mechoulam, 1966), cannabicyclolic (CBL), and cannabicyclolic acid (CBLA) through irradiation or decarboxylation (Shoyama et al., 1972). CBDA is the precursor of CBD, and THCA is the acidic precursor of Δ^9^-THC. THCA can be converted to Δ^8^-THC, cannabinol (CBN), and cannabinolic acid (CBNA) (Mechoulam and Gaoni, 1965; Elsohly and Slade, 2005).

### Terpenes

Terpenes are a large class of organic molecules responsible for flower aroma; they include β-caryophyllene, limonene, and linalool, which are present in 50 to 70% of all studied plants (Knudsen et al., 2006; Booth et al., 2017). For monoterpene biosynthesis, GPPS condenses one unit of IPP and DMAPP to form GPP, and GPP is converted into monoterpene form *via* mono-terpene synthase (TPS). Sesquiterpene biosynthesis requires two units of IPP to be added to a DMAPP unit. This sequential modification of DMAPP is ensured by farnesyl diphosphate synthase (FPPS) (Kulkarni et al., 2013). FPP is converted into sesquiterpenes *via* sesqui-TPS (Booth et al., 2017). Involvement of other enzymes such as cytochrome P450s leads to more complex terpenes (diterpenes, C_20_) (Grof, 2018; Booth and Bohlmann, 2019).

Independent of the inflorescence stage, major monoterpenes found in indoor-grown *C. sativa* L. “Finola” are α-pinene, β-pinene, β-ocimene, limonene, myrcene, and terpinolene (Booth and Bohlmann, 2019). Major sesquiterpenes expressed in trichomes are α-humulene, β-caryophyllene, bergamotene, and farnesene. As inflorescence matures, monoterpene accumulation increases relative to sesquiterpenes (Figure 1) (Booth et al., 2017). Although more than 120 terpenes have been identified in *C. sativa* L., many (including corresponding TPS genes) require further characterization (Aizpurua-Olaizola et al., 2016; Booth and Bohlmann, 2019). Since robust analytical standards are lacking, reported terpene profiles in *C. sativa* L. may contain some unknown terpene compounds, especially sesquiterpenes. A recent study reported more than 30 different TPS genes in the “Purple Kush” genome, and only 9 of 30 have been characterized (Günnewich et al., 2007; Booth et al., 2017). Elucidation of the underlying mechanisms surrounding terpene biosynthesis in cannabis plants may lead to further exploration and different medical applications for this PSM group (Aliferis and Bernard-Perron, 2020).

Terpenoids (a modified class of terpenes with different functional groups) are by far the most diverse group, with at least 80,000 different compounds (Christianson, 2017; Zhou and Pichersky, 2020). In recent years, cannabis terpenoids have slowly gained interest (Arena et al., 2016; Booth et al., 2017; Mudge et al., 2019). Studies have reported that terpenoids are powerful metabolites that have an interactive effect (or an “entourage effect”) with cannabinoid receptors (Gertsch et al., 2008). However, terpene composition in cannabis resin is dependent upon genetic, environmental, and developmental factors, and highly variable terpene profiles additionally exist between individual plants (Fischedick et al., 2010; Hazekamp and Fischedick, 2012; Booth et al., 2017). Terpene diversity in cannabis resin is responsible for scent and flavor qualities of cannabis flowers (Booth et al., 2017).

### Flavonoids

Members of the phenol family, flavonoids, form an important PSM group that aids in the plant's responses to sunlight and UV radiation (Downey et al., 2006; Warner et al., 2021). More than 20 flavonoid types in *C. sativa* L. have been identified, such as quercetin and kaempferol (Brenneisen, 2007). Others, such as cannflavins A, B, and C, are uniquely found in cannabis (Barrett et al., 1985, 1986; Radwan et al., 2008). Cannabis-specific flavonoids show promising therapeutic effects because of their anti-inflammatory activities (Barrett et al., 1985, 1986).

**Figure 2:**
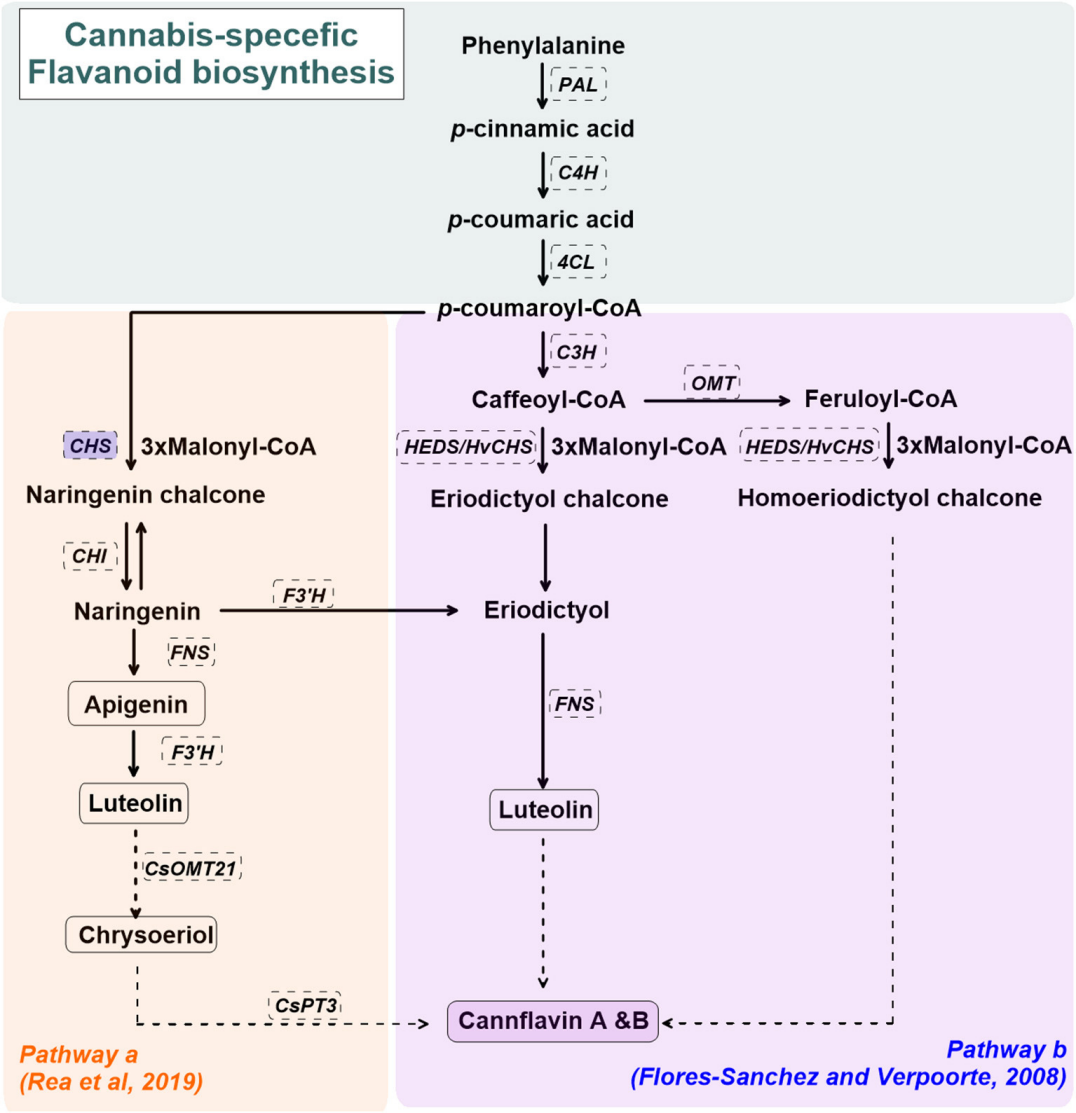
A simplified overview of the cannabis flavonoids, cannflavin A&B, pathway(s) in cannabis (*Cannabis sativa* L.), derived from Flores-Sanchez and Verpoorte (2008b) and Rea et al. (2019). Enzymes are in dashed line box. Enzymes in shaded blue boxes are upregulated by UV radiation in *Arabidopsis thaliana*. Dashed arrows represent proposed enzymatic reactions. CHS, chalcone synthase; CHI, chalcone isomerase; CsOMT21, *C. sativa* L. O-methyltransferase 21; CsPT3, *C. sativa* L. prenyltransferase 3; C4H, cinnamate 4-hydroxylase; C3H, p-coumaroyl-CoA 3-hydroxylase; FNS, flavone synthase; F3'H, flavonoid 3'-hydrolase; HEDS or HvCHS, homoeriodictyol/eriodictyol synthase; OMT, SAM-methyltransferase; PAL, phenylalanine ammonia-lyase; 4CL, 4-Coumarate:CoA ligase.

Cannabis-specific flavonoid biosynthesis is not well-established. Figure 2 shows the proposed biosynthetic pathway(s) for cannflavin A and B in *C. sativa* L. (Flores-Sanchez and Verpoorte, 2008b; Rea et al., 2019). The general pathway for cannflavin biosynthesis begins with *p*-coumaroyl-CoA derived from phenylalanine, phenylalanine ammonia-lyase (PAL), cinnamate 4-hydroxylase (C4H), and 4-Coumarate:CoA ligase (4CL). *p*-coumaroyl is covered to luteolin and cannflavin A and B via regiospecific methylation and prenylation reactions (Rea et al., 2019). Alternate routes for cannflavin A/B biosynthesis, beginning with feruloyl-CoA or caffeoyl-CoA with 3 malonyl-CoA, are also proposed (Flores-Sanchez and Verpoorte, 2008b). Although it has not been reported in *C.sativa* L., upregulated chalcone synthase (CHS) gene expression is observed in several plant species under abiotic stress such as UV radiation, as well as biotic stressors such as bacterial or fungal infection (Lipphardt et al., 1988; Dao et al., 2011).

Unlike cannabinoids and terpenes, flavonoid spatial and temporal distribution in cannabis plants does not follow the same pattern (Aizpurua-Olaizola et al., 2016). Rather, higher flavonoid content is reported in *C. sativa* L. leaves than other plant tissues (Flores-Sanchez and Verpoorte, 2008a; Jin et al., 2020). Apart from this, flavonoid concentration seems to decrease with plant tissue age (both leaves and inflorescence), in which higher flavonoid content is observed in young cannabis plants (Flores-Sanchez and Verpoorte, 2008a; Drinić et al., 2018). Low flavonoid content in cannabis oil and seeds is reported (Frassinetti et al., 2018; Moccia et al., 2019; Siano et al., 2019), while flavonoids are absent in glandular trichomes (Flores-Sanchez and Verpoorte, 2008b).

Recent studies show that flavonoid accumulation in inflorescence is variety-dependent and could be an indicator of the susceptibility of the variety to oxidative stress (Pavlovic et al., 2019; Giupponi et al., 2020). Pavlovic et al. (2019) reported the hemp variety “Futura 75” had higher cannabispiran concentration than “Finola.” This variety-dependent response is displayed elsewhere, where the hemp variety “Carmagnola Cs” has up to 25% more total phenol content (TPC) than other varieties, such as “Kompolti” (Izzo et al., 2020). Harnessing the radical scavenger activity and screening ability of flavonoids against UV radiation is a promising means of increasing flavonoid production in medical varieties (Agati and Tattini, 2010). Although it is out of the scope in this review, it is still worth to mention that the differences in the flavonoid quantifying methodologies, such as solvents used, matrix to solvent ratio, and characterization methods may result in flavonoid concentration discrepancies (Drinić et al., 2018; Frassinetti et al., 2018; Pellati et al., 2018).

## The Impact of Light Spectrum on Cannabis PSM Production

Plants respond to light stress by producing and accumulating PSM (Thirumurugan et al., 2018). The impact of UV radiation (>380 nm) and the visible light spectrum (380–740 nm) on PSM in greenhouse-grown crops has been well-studied (Urban et al., 2016; Gupta et al., 2017; Alrifai et al., 2019). However, the specific effects of light, including light properties (wavelength and intensity) and fixture configuration (i.e., overhead and subcanopy lighting) on cannabis PSM and phytochemistry is limited and not well-understood (Andre et al., 2016). These studies primarily focused on PSM accumulation in leaves rather than floral biomass. Table 1 summarizes available studies aimed at determining the impact of light spectrum and lighting configurations on cannabinoid and terpene accumulation.

**Table 1:** A comparison of cannabis PSM yield data compiled with overhead, subcanopy, or supplemental lighting.

	**Wavelength**		**Light intensity**	**Increased PSM**	**References**
	**Treatment**	**Control**			
Cannabinoids	Supplemental UV-B radiation	Mercury-vapor lamp and sunlight^a^	6.7 and 13.4 kJ m^−2^	Δ^9^-THC	Lydon et al., 1987
	Subcanopy 440+660 nm	440+660 nm^a^	50–500 μmol·m^−2^·s^−1^	CBGA and Δ^9^-THC	Hawley, 2018
	Subcanopy 440+530+660 nm	440+660 nm^a^	50–500 μmol·m^−2^·s^−1^	CBGA and Δ^9^-THC	Hawley, 2018
	410, 460, 540 +670 nm^a^	HPS^a^	450 μmol·m^−2^·s^−1^	CBD, CBG, Δ^9^-THC, and THCV	Magagnini et al., 2018
	450+630 nm^a^	HPS^a^	450 μmol·m^−2^·s^−1^	CBD and Δ^9^-THC	Magagnini et al., 2018
	~450+650 nm^a^, ^F^ (high blueand low red)	HPS^a^, ^F^	90 μmol·m^−2^·s^−1^	CBGA and Δ^9^-THC	Namdar et al., 2019
	Solar radiation (1,200 m ASL)	Solar radiation (130 m ASL)	–	CBDA	Giupponi et al., 2020^b^
	Full-spectrum LEDs	HPS	900 μmol·m^−2^·s^−1^	No impacts	Westmoreland et al., 2021^b^
Terpenes	Subcanopy 440+660 nm	440+660 nm^a^	50–500 μmol·m^−2^·s^−1^	*cis*-nerolidol	Hawley, 2018
	Subcanopy 440+530+660 nm	440+660 nm^a^	50–500 μmol·m^−2^·s^−1^	**Upper canopy:** α-pinine, limonene, myrcene, linalool, and cis-nerolidol **Lower canopy:** α-pinine, borneol, and *cis*-nerolidol	Hawley, 2018
	~450+650 nm^a^, ^V^ (high blueand low red)	Fluorescent lamp^a^, ^V^	180–200 μmol·m^−2^·s^−1^	Total terpene	Namdar et al., 2019
	Solar radiation (1,200 m ASL)	Solar radiation (130 m ASL)	–	β-myrcene, α-/β-pinene and limonene	Giupponi et al., 2020^b^

a *Overhead lighting;*

b *fiber-type cannabis (hemp); ASL, above sea level; CBD, cannabidiol; CBG, cannabigerol; CBGA, cannabigerolic acid; F, flowering stage; Δ^9^-THC, Δ^9^-tetrahydrocannabinol; THCV, tetrahydrocannabivarin; V, vegetative stage*.

### UV Radiation and PSM

Different wavelength ranges in UV radiation result in varying cannabinoid accumulation (Lydon et al., 1987; Magagnini et al., 2018). It has been nearly four decades since the first study suggesting that UV-B (280–315 nm) radiation affects cannabinoid accumulation in cannabis plants (Lydon et al., 1987). UV-B radiation did not impact cannabinoid content in both drug- and fiber-type cannabis plants, with the exception of Δ^9^-THC in bud tissues of drug-type cannabis plants. When the daily dosage of UV-B radiation increased from 0 to 13.4 kJ m^−2^, the Δ^9^-THC content increased from 25 to 32% (Lydon et al., 1987), suggesting that Δ^9^-THC was a UV-B photo-protectant (Pate, 1994). It was further noted that UV-B radiation increases trichome numbers. Altitude may be equally important. Increased solar UV radiation results in higher CBDA, terpene, and cannaflavin content in the hemp variety “Kompolti” (Giupponi et al., 2020). Notably, UV radiation sources used in both studies had relatively broad spectra, compared to electrical UV radiation sources, such UV-discharge lamps and light-emitting diodes (LEDs). It is unknown if there is was an interactive effect between UV-A (315–380 nm) and UV-B radiation, as a high percentage of UV-A radiation was present in both the UV-B and control light treatments (Mirecki and Teramura, 1984; Lydon et al., 1987; Giupponi et al., 2020). A subsequent study examined the impact of UV-A radiation on cannabinoid accumulation, and reported increased cannabinoid levels other than Δ^9^-THC (Magagnini et al., 2018). Low percentages of UV-A radiation (2%) from full-spectrum LED arrays induced an increase of several cannabinoids, including CBD, CBG, Δ^9^-THC, and tetrahydrocannabivarin (THCV), compared to a high pressure sodium (HPS) lamp that contained 1% of UV-A radiation (Magagnini et al., 2018). Clearly, more studies are required to clarify the impact of UV radiation on cannabis PSM accumulation.

### Visible Light and PSM

The impact of visible light on cannabis PSM accumulation has been investigated with different lighting configurations and different wavelengths (Hawley, 2018; Magagnini et al., 2018; Namdar et al., 2019) (Table 1). A high percentage of blue light cause increased cannabinoid content in cannabis inflorescence (drug-type cannabis, high amount of THC) (Hawley, 2018; Namdar et al., 2019; Danziger and Bernstein, 2021). Hawley (2018) examined the impact of subcanopy lighting with two different light spectra, 440 + 660 nm (blue + red, BR) and 440 + 530 + 660 nm (blue+ green + red, BGR), on cannabinoid and terpene accumulation. Increased Δ^9^-THC content and high CBGA levels were observed under both subcanopy BR and BGR lighting. Subcanopy BGR lighting had a higher impact on terpene accumulation than BR lighting, on both upper and lower canopies (Hawley, 2018). Increased CBGA content under LED lighting was similarly reported (Namdar et al., 2019; Danziger and Bernstein, 2021). During the flowering stage, light treatment with rich-blue light from overhead blue-red LED fixtures increased CBGA content and the CBGA: THCA ratio (Namdar et al., 2019).

Conflicting results on the interactive effects between blue light and cannabinoid content, however, were reported recently in fiber-type cannabis (hemp) (Wei et al., 2021; Westmoreland et al., 2021). Westmoreland et al. (2021) investigated the impact of light spectra on fiber-type cannabis and reported that neither CBD nor THC accumulation was impacted by spectral quality. The authors reported that this was likely caused by high light level (900 μmol·m^−2^·s^−1^) used as the saturation state of photoreceptors was reached, resulting in low sensitivity of cannabinoid accumulation to spectral quality (Westmoreland et al., 2021). Wei et al. (2021) also reported that no significant correlation between blue light fraction and cannabinoid yield was found in fiber-type cannabis; however, note that in this study the light levels used was between 28 and 540 μmol·m^−2^·s^−1^. As such, it is unknown that whether such variation on the interactive effect between spectral quality and cannabinoid accumulation is caused by light levels or cannabis chemotypes. Apart from blue light, supplemental green light induced cannabis PSM accumulation, including Δ^9^-THC and terpenes (limonene, linalool, and myrcene) (Hawley, 2018). No physiological theories explain how supplemental green light induces cannabis PSM accumulation. Clearly, both spectral properties and cannabis chemotype used highly impact cannabinoid accumulation, and further investigation on the links between spectral properties, cannabis chemotype, and photoreceptor is required to clarify the spectral effects.

## Photobiology and Molecular Pathways in *C. sativa* L. PSM Biosynthesis

Light regimes are elemental to *C. sativa* L. cultivation, as different wavelengths of light activate various light-dependent responses and related gene expression *via* photoreceptors and enzymes (Eichhorn Bilodeau et al., 2019; Aliferis and Bernard-Perron, 2020). Although the studies on cannabis growth and photobiology has expanded in the last few years, a comprehensive review by Aliferis and Bernard-Perron (2020) concludes that how light spectra influence cannabis metabolomics is still largely unknown. In particular, how cannabis PSM biosynthesis is impacted by monochromatic light requires further investigation, as most studies to date were conducted under mixed wavelength or full-spectrum light conditions.

**Figure 3:**
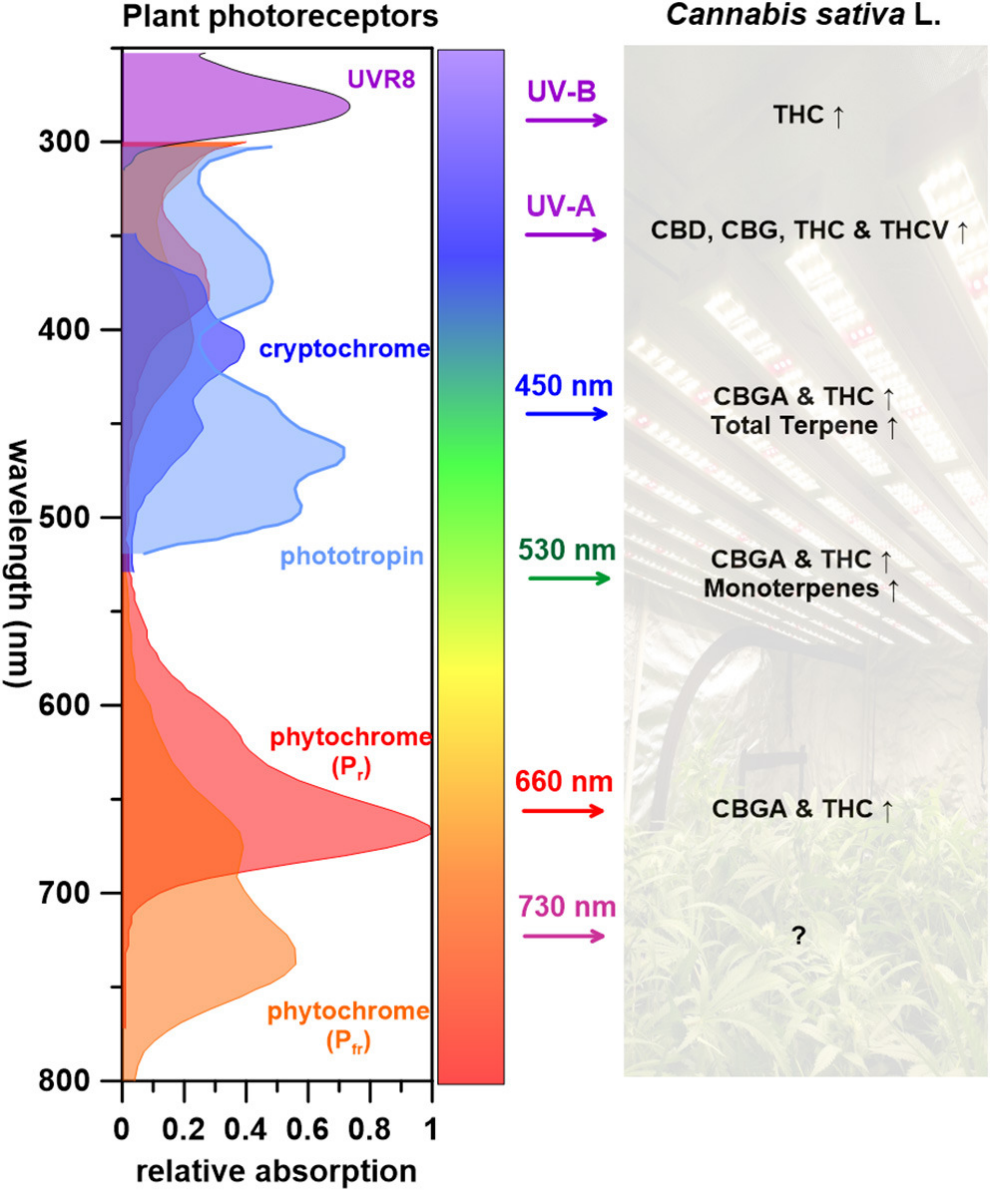
The impact of wavelengths on *Cannabis sativa* L. PSM responses, with corresponding photoreceptors (↑: increase, Δ: varying depended on light treatments, ↓: decrease, ?: unknown).

Figure 3 summarizes what is known of wavelengths and corresponding *C. sativa* L. PSM responses. UV radiation, one of the most effective wavelength ranges that induces cannabinoid biosynthesis (THC, THCV, CBD, and CBG), is perceived by several photoreceptors including UVR8, cryptochromes, and phototropins (Sager et al., 1988; Galvão and Fankhauser, 2015). Few studies have attempted to identify the regulatory elements of PSM biosynthetic pathway in cannabis plants (Marks et al., 2009; Bassolino et al., 2020). Some candidate regulatory genes for both cannabinoid and flavonoid biosynthesis(s) have been pinpointed and regulatory proteins identified; CsMYB77 and CsMYB94 for cannabinoid biosynthesis and CsbHLH112 and CsbHLH113 for flavonoid biosynthesis (Bassolino et al., 2020). Both MYB and bHLH superfamilies play key roles in the regulation of secondary metabolism (Hong, 2016). Follow up studies are required to place these cannabis proteins in the cannabinoid and flavonoid metabolic pathways. As for terpenes, although several studies indicate that UV-B radiation effects higher monoterpene content in plants that contain glandular trichomes (Johnson et al., 1999; Maffei and Scannerini, 2000), this has not yet been reported in *C. sativa* L. to our knowledge.

It has been proposed that light-dependent reactions for photosynthesis occur and supply energy for metabolic activity in tomato (*Solanum lycopersicum*) type VI glandular trichomes (Balcke et al., 2017). Using this as a precedence, it may be of interest to evaluate the global carbon and energy balance in *C. sativa* L. with different wavelengths of light to further elucidate trichome productivity and phytochemistry. Visible light (450, 530, and 660 nm) leads to increased CBGA, THC and terpene contents in *C. sativa* L. (Figure 3). When shifting wavelengths from UV radiation to the visible spectrum, cannabinoid precursor CBGA levels increases, yet no impact on THC is observed (Hawley, 2018; Namdar et al., 2019). Although Veress et al. (1990) reported that CBGA conversion to cannabinoids are non-enzymatic catalyzed reactions that naturally occurring postharvest during storage or heating (decarboxylation), it appears that light wavelengths can impact specific cannabinoid potency.

How visible light affects terpene biosynthesis remains elusive due to limited studies and terpene diversity (monoterpenes, sesquiterpene, and diterpenes). Drawing from previous studies of other crops may provide some insight and future direction for cannabis terpene production (Kessler and Kalske, 2018). When grown under blue LEDs, sage (*Pervoskia abrotanoides*, from the *Lamiaceae* family), sees its relative monoterpene content increase 3-fold upon exposure, with notable increases of α-thujene, α-pinene, and β-pinene. It was concluded that blue light could generally promote monoterpene content in *P. abrotanoides*, while augmented production of only one monoterpene, limonene, was observed in *P. atriplicifolia*. In this species, red light increased β-myrcene and cis-ocimene content (Ghaffari et al., 2019).

A nascent legal industry with proprietary value slows access to reliable information on indoor cannabis production, postharvest practices and processing of cannabis and cannabis-derived products. Apart from controlling environment (light, temperature, nutrients, microbiome etc.) to boost plant phytochemistry, optimal use of light pre- and post-harvest should be considered. For example, UV radiation could be used at the end of the flowering stage or before harvest to increase PSM production. More studies on how light can be manipulated during plant production and post-harvest for consistent PSM production and accumulation are anticipated.

## Concluding Remarks

Here we review known aspects of photobiology that are relevant to PSM production in *C. sativa* L., as cannabis research and development efforts are shifting from plant yield performance to manipulating cannabinoid, terpene, and flavonoid content. It is clear that light spectra can be manipulated to target specific cannabis PSM accumulation in different cannabis tissues (leaves and buds), resulting in altered potencies. Practically applied, optimized light regimes should reduce necessary electrical inputs while increasing cannabis PSM yields and quality. UV radiation is a powerful tool for stimulating cannabinoid biosynthesis in cannabis trichomes, while visible light alone impacts specific cannabinoid biosynthesis pathways and PSM profiles. UV radiation impacts terpene biosynthesis in other model plants, and this could be useful for cannabis plants. We expect that UV and blue LEDs will be increasingly used to stimulate desirable cannabis PSMs, as they have been widely applied and tailored to other high-value crops. The majority of cannabis studies are conducted under blue- and red-light mixtures, leaving a large sum of wavelengths in the visible spectrum untouched. Current evidence indicates that visible LED light can enhance CBG, THC, and terpene accumulation, but this is not explicitly seen with CBD. Gene regulatory and molecular pathways affecting cannabis metabolomics under monochromatic light remain elusive. Lighting strategies such as subcanopy lighting and varying light spectra for different plant growing stages and plant architecture can lower energy consumption and optimize cannabis PSM production, eventually improving the precision of cannabis PSM production, as well as therapeutic capacities.

Based on research reviewed, a few experimental directions are proposed to bridge knowledge gaps in cannabis lighting and PSM accumulation research: (1) The impact of narrow-spectrum light on cannabis PSM accumulation. Light spectrum greatly impacts cannabis PSM accumulation, yet there is minimal research available on the impact of narrow-spectrum light as most studies were conducted under either dichromatic or full-spectrum lighting. (2) Further investigations into the impact of high light in drug-type cannabis growth and its PSM accumulation, as our current knowledge in cannabis lighting is based on experimentation conducted under 500 μmol·m^−2^·s^−1^. (3) The impact of pre-harvest UV radiation treatment on cannabis PSM accumulation. UV LED sources with different wavelengths are highly available, and the accessibility to both researchers and producers make results more accessible.

## Author Contributions

VD and B-SW led the writing of this paper. B-SW and SM were the major editors. SM, VM, and ML contributed over 50% of the writing for the paper. ML is the correspondence point person. All authors contributed to the article and approved the submitted version.

## Conflict of Interest

The authors declare that this study received funding from EXKA Inc. The funder was not involved in the study design, collection, analysis, interpretation of data, the writing of this article or the decision to submit for publication.

## Abbreviations

CBC: cannabichromene
CBCA: cannabichromentic acid
CBCAS: cannabichromentic acid synthase
CBDAS: cannabidiolic acid synthase
CBD: cannabidiol
CBDA: cannabidiolic acid
CBG: cannabigerol
CBGA: cannabigerolic acid
CBL: cannabicyclol
CBLA: cannabicyclolic acid
CBN: cannabinol
CBNA: cannabinolic acid
CHS: chalcone synthase
CHI: chalcone isomerase
CsOMT21: *C. sativa* L. O-methyltransferase 21
CsPT3: *C. sativa* L. prenyltransferase 3
C4H: cinnamate 4-hydroxylase
C3H: p-coumaroyl-CoA 3-hydroxylase
DMAPP: dimethylallyl pyrophosphate
DXS: 1-deoxy d-xylulose-5-phosphate synthase
DXP: 1-deoxy-D-xylulose 5- phosphate
FNS: flavone synthase
FPP: farnesyl diphosphate
FPPS: farnesyl diphosphate synthase
F3'H: flavonoid 3'-hydrolase
G3P: glyceraldehyde 3-phosphate
GPP: geranyl pyrophosphate
GPPS: geranyl pyrophosphate synthase
HEDS or HvCHS: homoeriodictyol/eriodictyol synthase
HPS: high pressure sodium
IPP: isopentenyl diphosphate
IPPi: isopentenyl-diphosphate delta-isomerase
LED: light-emitting diode
LS: limonene synthase
MEP: methylerythritol phosphate
MEV: mevalonate
OA: olivetolic acid
OAC: olivetolic acid cyclase
OMT: SAM-methyltransferase
PAL: phenylalanine ammonia-lyase
PSM: plant secondary metabolite
PT4: geranylpyrophosphate: olivetolate geranyltransferase 4
Δ^8^-THC: Δ^8^-tetrahydrocannabinol
Δ^9^-THC (or THC): Δ^9^-tetrahydrocannabinol
THCA: tetrahydrocannabinolic acid
THCAS: tetrahydrocannabinolic acid synthase
THCV: tetrahydrocannabivarin
TPS: terpene synthase
TK: tetraketide
TKS: tetraketide synthase
UV: ultraviolet
4CL: 4-Coumarate:CoA ligase.
